# Synthesis, Surface and Antimicrobial Activity of Piperidine-Based Sulfobetaines

**DOI:** 10.1007/s11743-016-1906-8

**Published:** 2016-11-15

**Authors:** Daria Wieczorek, Adam Dobrowolski, Katarzyna Staszak, Dobrawa Kwaśniewska, Patrycja Dubyk

**Affiliations:** 1 Department of Technology and Instrumental Analysis, Faculty of Commodity Science, Poznan University of Economics and Business, al. Niepodległości 10, 61-875 Poznan, Poland; 2 Department of Biotechnology and Food Microbiology, Faculty of Food Science, Wrocław University of Environmental and Life Sciences, ul. Chełmońskiego 37/41, 51-630 Wroclaw, Poland; 3 Institute of Technology and Chemical Engineering, Poznan University of Technology, ul. Berdychowo 4, 60-965 Poznan, Poland

**Keywords:** Sulfobetaines, Interfacial activity, Biological properties

## Abstract

A new method for the preparation of new heterocyclic amine surfactants based on sulfobetaines is proposed. Interfacial activities of the surfactants obtained in aqueous solution were studied by surface tension measurements. The critical micelle concentration, surface excess concentration, minimum area per surfactant molecule, and standard Gibbs energy of adsorption were determined. The adsorption properties of these compounds depend significantly on the alkyl chain length. Alkyl chain length also affects biological properties of the new surfactants, determining the minimum inhibitory concentration and size of inhibited growth zone. The compounds have high antimicrobial activity.

## Introduction

Although amphoteric materials represent only a small portion of total worldwide surfactant production, their market position is increasing significantly because of their unique properties. Their nature can make them especially useful in applications requiring biological contact [1, 2]. The group of amphoteric surface active agents is represented by zwitterionic surfactants. Their characteristic features are a consequence of the structure as their molecules carry both negative and positive charge [1, 3]. The molecular structure of these surfactants, in particular the alkyl chain length, number of hydrophobic chains, nature and number of head groups and structure of the spacer between positively and negatively charged moieties, strongly affects their physicochemical and biological properties [4]. For most zwitterionic surfactants, the cationic moiety consists of a cationic quaternary ammonium group, while the anionic moiety includes a carboxylic acid, sulfonic acid, sulfuric acid ester, or phosphoric acid ester [5].

Zwitterionic molecules of the sulfobetaine type are applied in different fields of chemistry [6]. These compounds are used in production of modified polymers, e.g., poly(sulfobetaine methacrylate). The coexistence of positive and negative charge on the surfactant molecule generates a hydration layer as a result of strong electrostatic interactions. Super low fouling properties of zwitterionic materials such as poly(sulfobetaine methacrylate) arise from this hydration layer, contributing to the reduced protein adsorption, cell attachment, and bacterial adhesion [7]. Polymers incorporating zwitterionic sulfobetaines have been recognized as promising candidates for responsive systems geared towards various potential applications such as biosensors, catalysts, drug delivery systems, and separation media [8–10]. Moreover, sulfobetaine surfactants can be used as antimicrobial agents [11, 12].

Sulfobetaines differ from each other in the length of and the presence of hydroxyl groups in the spacer separating the quaternary ammonium center from the sulfonate group. Besides different spacers, the amines used for the synthesis of these surfactants can also be different [13].

The significant interest in zwitterionic surfactants prompted us to prepare a series of sulfobetaine surfactants using a piperidine moiety with N-alkyl substituents of variable chain length (C10–C16) with an N-alkyl C3 or C4 spacer with a terminal sulfonate group. We aimed to determine surface activities by study of surface tension. Additionally, microbial activity against both Gram positive and Gram negative bacteria and one yeast species was examined. The effect of the chemical structure (alkyl length chain or length of spacer between quaternary ammonium center and sulfonate group) of these surfactants on their properties is discussed.

## Materials and Methods

### Synthesis Procedures

In the first step of *N*-alkylpiperidine synthesis, piperidine (0.04 mol) was reacted with alkyl bromide (0.02 mol). The reaction was carried out for several hours at room temperature using diethyl ether as solvent. The resulting precipitate was filtered off, while excess solvent was evaporated from the solution. Liquid piperidine derivatives with 10-, 12-, 14-, and 16- carbon chains were obtained.

**Fig. 1:**
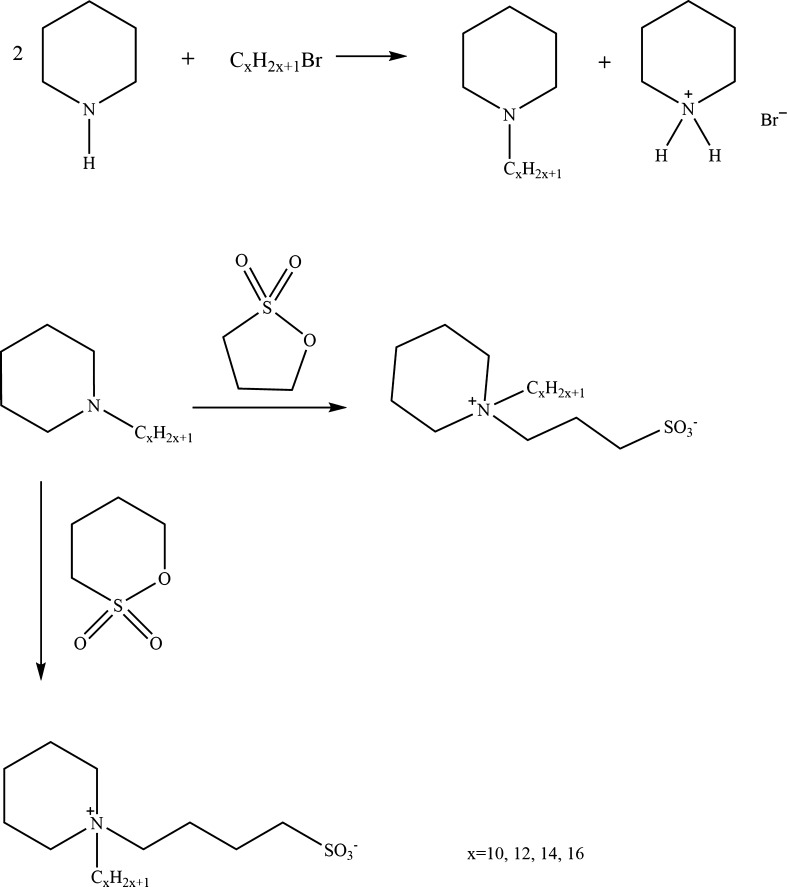
Synthesis route

In the next step, to get *N*-alkyl-*N*-(propylpiperidinium-3-sulfate) or *N*-alkyl-*N*-(butylpiperidinium-4-sulfate), the 1,3-propane or 1,4-butane sultone (0.1 mol), respectively, was dissolved in ethyl acetate and *N*-alkylpiperidine (0.1 mol) was then added. The mixture was left for several days with protection against ambient moisture. The product was filtered off and the crude product was recrystallized from methanol/ethyl acetate. The reaction scheme is shown in Fig. 1.

### Synthesis Results

All synthesized surfactants and their abbreviations are presented in Table 1. The structures of the obtained compounds were confirmed by spectroscopic methods and elemental analysis and are presented below.

**Table 1:** Surface properties and adsorption parameters of surfactants

Surfactant	CMC (mmol/dm^3^)	γ_CMC_ (mN/m)	p*C*_20_ (mol/dm)	Π_CMC_ (mN/m)	Δ*G*_m_^0^ (kJ/mol)	*A*_Sz_ × 10^8^ (mol/dm^3^)	*B*_Sz_ × 10^2^	Γ^∞^ × 10^6^ (mol/m^2^)	Corr (–)	Δ*G*_abs_ (kJ/mol)	*A*_min_ × 10^19^ (m^2^)
P10S3	8.70	29.01	2.95	42.59	–21.43	2511	21.35	6.44	0.976	–20.3	2.58
P12S3	3.97	39.81	3.97	31.79	–23.35	1350	9.44	2.75	0.992	–27.4	6.03
P14S3	0.18	38.23	4.22	33.37	–30.91	84.78	8.58	2.59	0.995	–34.2	6.41
P16S3	0.005	36.66	5.66	34.93	–39.68	0.0012	3.28	0.99	0.994	–61.4	16.76
P10S4	9.78	23.95	2.73	47.65	–21.14	18,710	14.81	4.31	0.992	–21.0	3.85
P12S4	1.79	31.59	3.30	40.01	–25.29	2454	11.57	3.34	0.987	–25.7	4.98
P14S4	0.11	34.08	4.47	37.52	–32.12	39.89	7.91	2.30	0.994	–36.0	7.23
P16S4	0.002	31.93	6.19	39.67	–41.92	0.036	4.42	1.28	0.977	–53.2	12.95

#### *N*-Decyl-*N*-(propylpiperidinium-3-sulfate) (P10S3)

^1^H NMR (CDCl_3_) *δ* = 0.88 (m, 3H, CH_3_), 1.26 (m, 12H, CH_2_), 1.79 (m, 6H, 3CH_2_), 2.02 (s, 2H, CH_2_), 2.19 (s, 2H, CH_2_), 2.91 (s, 2H, CH_2_SO_3_^−^), 3.27 (m, 2H, CH_2_), 3.46 (m, 2H, CH_2_N^+^), 3.55 (m, 2H, CH_2_N^+^), 3.70 (m, 2H, CH_2_N^+^), 4.00 (m, 2H, CH_2_N^+^). ^13^C NMR (CDCl_3_) *δ* = 14.16, 18.1, 19.6, 20.8, 21.4, 22.6, 23.7, 26.4, 29.3, 31.8, 47.4, 53.1, 57.8, 59.1. IR = 1034, 1196, 2853, 2920 cm^−1^. Anal. Calcd: C, 62.25; H, 10.66; N, 4.03; S, 9.22. Found: C, 61.08; H, 10.53; N, 3.73; S, 8.63. mp 172–173 °C, yield 62%.

#### *N*-Dodecyl-*N*-(propylpiperidinium-3-sulfate) (P12S3)

^1^H NMR (CDCl_3_) *δ* = 0.88 (m, 3H, CH_3_), 1.26 (m, 14H, CH_2_), 1.35 (s, 2H, CH_2_) 1.70–1.84 (m, 6H, 3CH_2_), 1.98 (s, 2H, CH_2_), 2.17 (s, 2H, CH_2_), 2.91 (s, 2H, CH_2_SO_3_^−^), 3.26 (m, 2H, CH_2_), 3.46 (m, 2H, CH_2_N^+^), 3.53 (m, 2H, CH_2_N^+^), 3.69 (m, 2H, CH_2_N^+^), 4.49 (m, 2H, CH_2_N^+^). ^13^C NMR (CDCl_3_) *δ* = 13.9, 18.0, 19.6, 20.7, 21.3, 22.5, 23.5, 26.4, 29.4, 31.7, 47.4, 52.9, 58.0, 59.0. IR = 1033, 1177, 2851, 2919 cm^−1^. Anal. Calcd: C, 64.00; H, 10.93; N, 3.73; S, 5.53. Found: C, 63.37; H, 11.48; N, 3.43; S, 7.91. mp 174–175 °C, yield 56%.

#### *N*-Tetradecyl-*N*-(propylpiperidinium-3-sulfate) (P14S3)

^1^H NMR (CDCl_3_) *δ* = 0.88 (3H, CH_3_), 1.26 (m, 18H, CH_2_), 1.35 (s, 4H, 2CH_2_), 1.70–1.81 (m, 6H, 3CH_2_), 2.02 (s, 2H, CH_2_), 2.18 (s, 2H, CH_2_SO_3_^−^), 2.91 (m, 2H, CH_2_), 3.26 (m, 2H, CH_2_N^+^), 3.41 (m, 2H, CH_2_N^+^), 3.58 (m, 2H, CH_2_N^+^), 3.72 (m, 2H, CH_2_N^+^). ^13^C NMR (CDCl_3_) *δ* = 14.0, 18.3, 19.7, 20.9, 21.5, 22.6, 23.5, 26.5, 29.3, 31.8, 47.5, 49.9, 53.1, 57.8, 59.2. IR = 1035, 1197, 2853, 2920 cm^−1^. Anal. Calcd: C, 65.51; H, 11.17; N, 3.47; S, 7.94. Found: C, 64.58; H, 11.67; N, 3.22; S, 7.41. mp 173–174 °C, yield 48%.

#### *N*-Hexadecyl-*N*-(propylpiperidinium-3-sulfate) (P16S3)

^1^H NMR (CDCl_3_) *δ* = 0.88 (3H, CH_3_), 1.20 (m, 26H, CH_2_), 1.71–1.81 (m, 6H, 3CH_2_), 1.99 (s, 2H, CH_2_), 2.17 (s, 2H, CH_2_SO_3_^−^), 2.89 (m, 2H, CH_2_), 3.27 (m, 2H, CH_2_N^+^), 3.43 (m, 2H, CH_2_N^+^), 3.57 (m, 2H, CH_2_N^+^), 3.75 (m, 2H, CH_2_N^+^). ^13^C NMR (CDCl_3_) *δ* = 14.0, 18.3, 19.7, 20.8, 21.4, 22.6, 26.6, 29.4, 31.8, 47.4, 53.0, 57.8, 59.1. IR = 1035, 1165, 2853, 2920 cm^−1^. Anal. Calcd: C, 66.98; H, 11.16; N, 3.26; S, 7.44. Found: C, 65.42; H, 11.79; N, 2.90; S, 6.66. mp 165–166 °C, yield 35%.

#### *N*-Decyl-*N*-(butylpiperidinium-4-sulfate) (P10S4)

^1^H NMR (CDCl_3_) *δ* = 0.90 (m, 3H, CH_3_), 1.26 (m, 12H, CH_2_), 1.61 (4H, CH_2_), 1.92 (m, 8H, CH_2_), 2.28 (m, 2H, CH_2_SO_3_^−^), 2.87 (m, 2H, CH_2_), 3.19 (m, 2H, CH_2_N^+^), 3.30 (m, 2H, CH_2_N^+^), 3.46 (m, 2H, CH_2_N^+^), 3.65 (m, 2H, CH_2_N^+^). ^13^C NMR (CDCl_3_) *δ* = 13.9, 19.7, 20.2, 22.4, 23.5, 24.2, 25.7, 26.3, 27.5, 29.2, 31.6, 48.1, 50.0, 54.4, 58.8, 59.6. IR = 1034, 1184, 2855, 2926 cm^−1^. Anal. Calcd: C, 63.16; H, 10.80; N, 3.88; S, 8.86. Found: C, 61.47; H, 11.13; N, 3.50; S, 7.21. mp 194–195 °C, yield 48%.

#### *N*-Dodecyl-*N*-(butylpiperidinium-4-sulfate) (P12S4)

^1^H NMR (CDCl_3_) *δ* = 0.881 (m, 3H, CH_3_), 1.26 (m, 16H, CH_2_), 1.65 (m, 4H, CH_2_), 1.91 (m, 8H, CH_2_), 2.28 (m, 2H, CH_2_SO_3_^−^), 2.86 (m, 2H, CH_2_), 3.20 (m, 2H, CH_2_N^+^), 3.30 (m, 2H, CH_2_N^+^), 3.46 (m, 2H, CH_2_N^+^), 3.61 (m, 2H, CH_2_N^+^). ^13^C NMR (CDCl_3_) *δ* = 13.8, 19.6, 20.2, 22.4, 23.4, 24.3, 25.6, 26.2, 27.2, 29.3, 31.6, 48.0, 50.1, 53.8, 57.2, 58.7. IR = 1035, 1186, 2855, 2921 cm^−1^. Anal. Calcd: C, 64.78; H, 11.05; N, 3.60; S, 8.23. Found: C, 60.56; H, 10.78; N, 3.35; S, 7.53. mp 175–176 °C, yield 31%.

#### *N*-Tetradecyl-*N*-(butylpiperidinium-4-sulfate) (P14S4)

^1^H NMR (CDCl_3_) *δ* = 0.88 (m, 3H, CH_3_), 1.26 (m, 20H, CH_2_), 1.35 (m, 2H, CH_2_), 1.94 (m, 8H, CH_2_), 2.26 (m, 2H, CH_2_SO_3_^−^), 2.90 (m, 2H, CH_2_), 3.19 (m, 2H, CH_2_N^+^), 3.26 (m, 2H, CH_2_N^+^), 3.43 (m, 2H, CH_2_N^+^), 3.66 (m, 2H, CH_2_N^+^). ^13^C NMR (CDCl_3_) *δ* = 14.1, 19.8, 20.4, 22.6, 23.5, 24.1, 25.4, 26.5, 27.8, 29.5, 31.8, 48.3, 50.1, 54.4, 56.9, 59.9. IR = 1035, 1193, 2850, 2919 cm^−1^. Anal. Calcd: C, 66.19; H, 11.27; N, 3.36; S, 7.67. Found: C, 62.14; H, 10.95; N, 2.80; S, 8.39. mp 182–183 °C, yield 25%.

#### *N*-Hexadecyl-*N*-(butylpiperidinium-4-sulfate) (P16S4)

^1^H NMR (CDCl_3_) *δ* = 0.88 (m, 3H, CH_3_), 1.26 (m, 22H, CH_2_), 1.61 (m, 4H, CH_2_), 1.93 (m, 8H, CH_2_), 2.28 (m, 2H, CH_2_SO_3_^−^), 2.89 (m, 2H, CH_2_), 3.17 (m, 2H, CH_2_N^+^), 3.26 (m, 2H, CH_2_N^+^), 3.52 (m, 2H, CH_2_N^+^), 3.64 (m, 2H, CH_2_N^+^). ^13^C NMR (CDCl_3_) *δ* = 14.1, 19.8, 20.4, 22.6, 23.6, 24.2, 25.6, 26.5, 27.6, 29.6, 31.9, 48.3, 50.2, 54.4, 59.0, 59.6. IR = 1033, 1183, 2851, 2921 cm^−1^. Anal. Calcd: C, 67.57; H, 11.26; N, 3.15; S, 7.21. Found: C, 64.21; H, 11.40; N, 2.64; S, 7.19. mp 143 °C, yield 22%.

### Determination of Surface Activity

The surface tension of the aqueous solutions of surfactants was measured by the Du Noüy ring method with a K12 KRÜSS tensiometer, with resolution 0.01 mN/m, at a constant temperature of 21 °C. The deviation between three replicate measurements was in the range of 0.05–0.22 mN/m. Measurements were made for the aqueous solution at the initial concentration of 50 mM; other solutions were obtained by the serial dilution method.

### Microorganism and Culture Conditions

The antimicrobial activity of the surfactants was tested on the following strains of microorganisms: (1) Gram positive bacteria *Staphylococcus aureus* (ATCC 9538), *Bacillus subtilis* (ATCC 6633), and *Enterococcus hirae* (ATCC 10542); (2) Gram negative bacteria *Escherichia coli* (ATCC 10536) and *Pseudomonas aeruginosa* (ATCC 15442); (3) and yeast *Candida albicans* (ATCC 10231). All the microorganisms were obtained from culture collection of the Department of Biotechnology and Food Microbiology (UELS, Wrocław). The bacteria were grown in nutrient broth medium at 37 °C, except *B. subtilis* (ATCC 6633) which was grown at 30 °C. The yeast was grown in YPD medium at 30 °C. Agar was added to the medium at a concentration of 2% when necessary. The investigation of all three classes of microorganism allows a robust assessment of antimicrobial activity of these piperidine-based sulfobetaines.

### Determination of Antimicrobial Properties by Agar Diffusion Assay

Agar diffusion assay (well diffusion assay) was used for testing the antimicrobial activity of the newly synthesized surfactants. Wells were made in seeded agar and the test samples were introduced directly into these wells. After incubation, the distances between the edge of the well and the end of the clear zones around the wells were measured. Briefly, on a sterile Petri dish containing appropriate agar medium, 200 µL of bacterial or yeast overnight cultures washed in saline solution and adjusted to OD_600_ = 1 was applied (which corresponds to 6 × 10^6^ cells of yeast and 2 × 10^8^ cells of bacteria). Next, four wells per plate were made with a sterile Pasteur pipette (8.4 mm diameter). Subsequently, 100 µL of aqueous solution of surfactants (5 mg/mL) was poured into each well and incubated 6 h in 4 °C to achieve full diffusion of the solution tested in the agar medium. Next, prepared Petri plates were incubated at 30 °C or 37 °C for 48 h and zones of inhibited growth around each well were measured. On each plate, the three wells contained, respectively, a solution of surfactant, a control, and sterile saline solution. All assays were carried out three times. The standard deviation of all analyzed zones of the antimicrobial activity did not exceed 3%.

### Determination of Minimum Inhibitory Concentration (MIC)

The microdilution method was used to determine the minimum inhibitory concentrations (MICs) of the new synthesized surfactants. The experiments were performed in 100-well microplates (honeycomb) with use of a Bioscreen C microbial growth monitoring system (Oy Growth Curves Ab Ltd., Finland). Portions of 200 μL of an appropriate medium (nutrient broth for bacteria and YPD for yeast) containing different concentrations of one surfactant (0.01–5 mg/mL) were dispensed into the wells of a microplate. Growth control wells did not contain any surfactant. All wells (except blank controls for each concentration of each surfactant) were inoculated with 5 μL of overnight bacterial or yeast washed cultures (diluted to reach final OD_600_ = 0.1). The plates were incubated for 48 h at 30 or 37 °C under constant agitation. The growth of microorganisms was monitored by measuring optical density at 420−560 nm every 20 min, and the data were collected using Bioscreen C software. Assays were carried out three times in five replicates (five wells for each concentration of surfactant).

## Results and Discussion

### Surface Activity

**Fig. 2:**
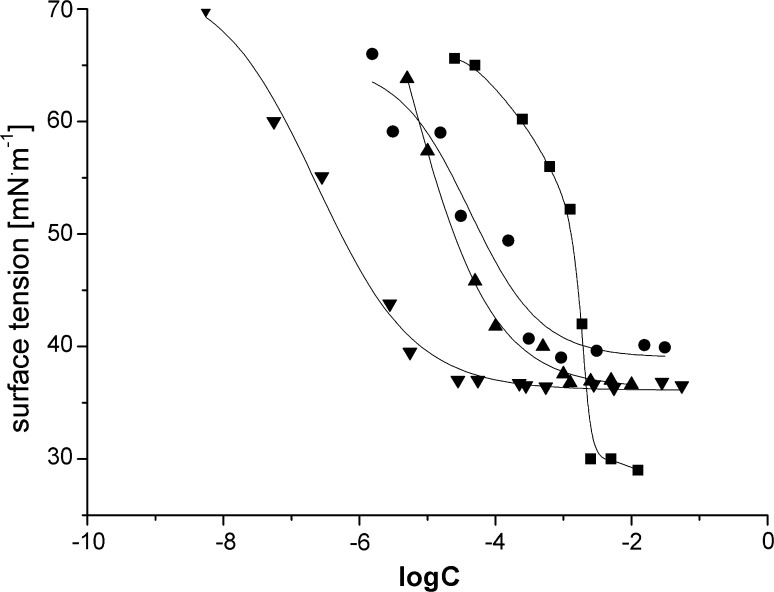
Surface tension isotherms for ■ P10S3, ● P12S3, ▲ P14S3, ▼ P16S3

**Fig. 3:**
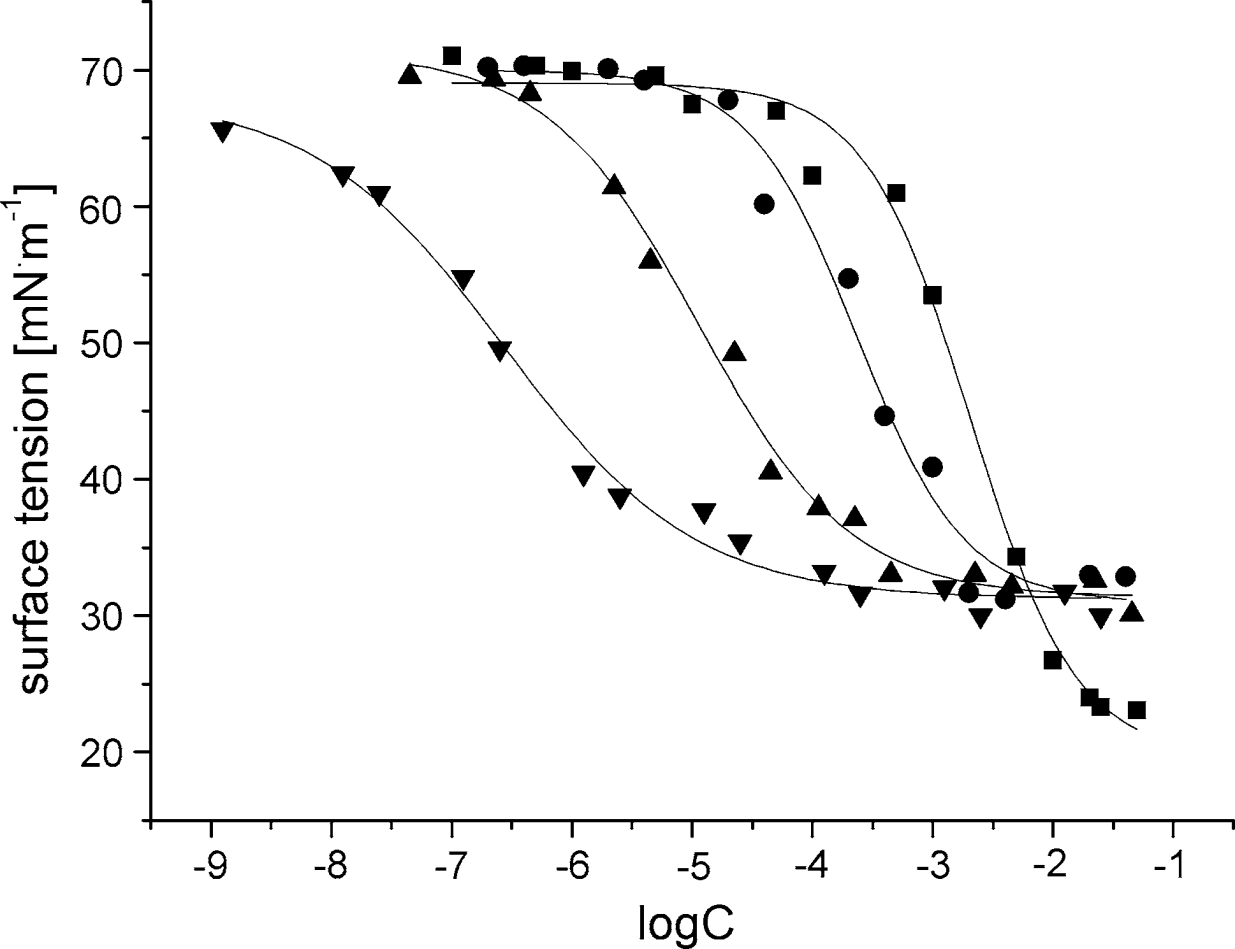
Surface tension isotherms for ■ P10S4, ● P12S4, ▲ P14S4, ▼ P16S4

The most important property of surfactants is the ability to lower surface and interfacial tension. Another interesting property of aqueous surfactant solution is the formation of micelles above the critical concentration (CMC), which can be determined from the experimental results of the surface tension for a series of aqueous solutions of different concentrations. Figures 2 and 3 presents adsorption isotherms of sulfobetaines studied. Their CMC values were determined from bilateral extrapolation of the straight sections of the isotherms, with results are presented in Table 1.

As shown in Table 1, the CMC values decrease with increasing alkyl chain length. The relationship between the number of carbon atoms in the alkyl chain (*n*) and CMC can be expressed by the following equation:

1 $${ \log }\left( {\text{CMC}} \right) = A{-}B\;{ \times }\;n ,$$

where *A* is a constant characteristic of a particular ionic head at a given temperature and *B* depends on the type of surfactant. For cationic and anionic surfactants *B* is close to 0.3 [14], for nonionic [15] and zwitterionic ones it is 0.5 [5]. The values of *B* for *N*-alkyl-*N*-(propylpiperidinium-4-sulfate) and *N*-alkyl-*N*-(butylpiperidinium-4-sulfate) are equal to 0.55 and 0.61, respectively. Thus, the behavior of the surfactants studied seems to be similar to that in past studies of zwitterionic surfactants.

Moreover, from the results presented in Table 1 it can be concluded that the nominal values of CMC are higher for propane derivatives than for butane ones. This can be explained by an increase in their hydrophobicity. The same observations were made by Staszak *et al*. for sulfobetaines with morpholinium moiety [13].

It is hard to find a similar correlation between CMC and the length of the spacer. For instance, Cheng *et al.* [16] studied amido-amine-based cationic gemini surfactants with propyl and hexyl spacer groups and showed that derivatives of the compounds with longer alkyl chain in the spacer group had higher CMC values; however, the authors did not comment on the reasons for this phenomenon.

The reduction in surface tension depends on the replacement of solvent molecules with the surfactant ones at the interface. The efficiency of a surfactant in reducing surface tension should reflect its concentration at the interface relative to that in the bulk liquid phase and can be described by the parameters γ_CMC_, Π_CMC_, and p*C*_20_. The value of surface tension at CMC (γ_CMC_) is in the range of 29.0–39.8 and 25.4–34.1 mN/m for propane and butane derivatives, respectively. Thus the ability to reduce the value of surface tension at CMC, corresponding to the surface activity, is higher for butane derivatives than for the corresponding propane ones. There was no observation of alkyl chain length dependence of this ability.

The effectiveness (Π_CMC_) of a surfactant to reduce surface tension can be measured by the surface pressure, Π_CMC_ = γ_0_ – γ_CMC_, attained at the critical micelle concentration, since the reduction of the tension below CMC is relatively insignificant [3]. The higher the value of Π_CMC_ is, the more effectively the surface tension value is reduced. This effectiveness of butane derivatives was higher.

A convenient measure of the efficiency of adsorption is the negative logarithm of the concentration of surfactant in the bulk phase needed to produce 20 mN/m reductions in the surface tension of the solvent, p*C*_20_. High values indicate that surfactants more efficiently adsorb at the interface and reduce surface tension. As follows from data in Table 2, the p*C*_20_ values increase with increasing alkyl chain length for both homologous series. This fact indicates that greater reduction in surface tension is achieved for compounds with longer alkyl chain used at a smaller concentration. The same tendency was observed for sulfopropane betaines H-(CH_2_)_m_ N^+^(CH_3_)_2_(CH_2_)_3_SO_3_^−^ with *m* = 12, 14, 16, 18 and sulfobutane betaines H-(CH_2_)_n_ N^+^(CH_3_)_2_(CH_2_)_4_SO_3_^−^ with *n* = 12, 14, 16, 18 [17].

**Table 2:**
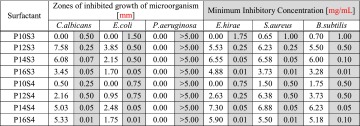
Zones of inhibited growth and MICs of the surfactants on the panel of tested microorganisms

The standard free energy of micellization (Δ*G*_m_^0^) was calculated from the equation

2 $$\Delta G_{\text{m}}^{ 0} = RT\ln X_{\text{CMC}} .$$

Moreover, the surface tension data were fitted to Szyszkowski’s equation [18] and the following parameters were estimated: the surface excess at the saturated interface (Γ_∞_), the minimum molecular area in the adsorption layer at the saturated interface (*A*_min_), and the Gibbs free energy of adsorption (Δ*G*_ads_), according to the equations [19]

3 $$\Gamma _{\infty } = \frac{{B_{\text{Sz}} \gamma_{0} }}{RT},$$

4 $$A_{\hbox{min} } = \frac{1}{{\Gamma _{\infty } N_{\text{A}} }},$$

5 $$\Delta G_{\text{ads}}^{\text{Sz}} = RT\ln (A_{\hbox{min} } ),$$

where *γ*_0_, *R*, *T*, and *N*_A_ stand for interfacial tension of solvent (here water), gas constant, temperature, and Avogadro’s constant, respectively. The values of correlation coefficients (cor) for the Szyszkowski isotherms, presented in Table 1, indicated a good fit of the experimental data to the proposed model.

The values of both free energies of adsorption and micellization decreased with increasing number of carbon atoms in the alkyl chain. However, the values for the C_10_ surfactants were more negative for Δ*G*_m_^0^, indicating that the compounds obtained exhibited greater tendency to form micelles than to adsorb at the water–air interface. With increasing length of the alkyl group the surfactant tendency to get adsorbed at the water–air interface is greater than that of micelle formation.

Estimated values of Γ_∞_ decrease and *A*_min_ increase with increasing aliphatic chain length in the surfactant molecules. Thus, the adsorption efficiency is higher for the surfactant molecules with shorter aliphatic chain. The results obtained indicate that the structure of adsorption monolayer depends on the hydrophobicity of the compounds studied. Molecules with short aliphatic chains are much more densely populated at the saturated air–water interface. These results are in contrast to those obtained for other betaine-type surfactants [13, 17, 20]. However, in a few papers no relationship between *A*_min_ and alkyl length chain has been observed [21] or like here *A*_min_ increased with increasing aliphatic chain length in the surfactant molecules [22–24].

### Antimicrobial Properties of Surfactants

Antimicrobial activities of the surfactants studied were first evaluated by the well diffusion method. The solutions of each surfactant added to the wells were found to inhibit the growth of almost all microorganisms tested, except the strain of *P. aeruginosa*, which was resistant to all surfactants (Table 2). The weakest effect was observed for surfactants P10S3 and P10S4 for which no inhibition was noted for four and three microorganisms, respectively, and very small zones were measured for the remaining microorganisms. The strongest effect was observed for surfactant P12S3 against *C. albicans* (7.58 mm) and for Gram positive bacteria with the largest zone for P14S4 surfactant when tested on *E. hirae* (7.3 mm), *S. aureus* (6.88 mm), and *B. subtilis* (6.23 mm). Gram negative bacteria *E. coli* was sensitive to all surfactants, except P10S3 and P10S4, but with much smaller zones of inhibited growth compared to those obtained for Gram positive bacteria (max zone for P12S3 = 3.85 mm). Also, we [12] reported a higher antibacterial activity of studied surfactants against Gram positive (*B. subtilis* and *S. aureus*) than Gram negative bacteria (*E. coli* and *P. aeruginosa*), which corresponds to the results obtained in this work [25]. Comparison of the sizes of inhibited growth zones determined for the surfactants studied with those evaluated for the surfactants used as disinfectants shows that the newly obtained sulfobetaines are much more active. The effectiveness of cetylpyridinium chloride (CPC) against *S. aureus* and *B. subtilis* measured as the size of the inhibition growth zone was 2.0 mm, while the zone measured for* E* *. coli* was 1.7 mm [26]. For more accurate study of antimicrobial activity of the newly synthesized surfactants the values of MICs for selected strains were determined by the microdilution method. The lowest MIC was observed for the surfactants P16S4, P16S3 (0.01 mg/mL) and P14S4, P14S3 (0.05 mg/mL) which indicates the highest antimicrobial activity. The highest MIC values were observed for P10S3 and P10S4, which indicates the weakest antimicrobial properties, which is in the agreement with previously described plate diffusion experiments. Increasing the alkyl chain length improved the antimicrobial properties. Such a phenomenon is unique and is related to the structure of the polar head, which has been confirmed by literature reports [27, 28]. The effect of alkyl chain length was briefly discussed for quaternary ammonium compounds (QACs) [29]. They are the most prevalent forms of cationic surfactants used today because they have a broad spectrum of microbiological activities over wide pH ranges and are used in industry, agriculture, hospitals, and housekeeping. The highest antimicrobial activity of a homologous series (C12–C16) of alkyldimethylbenzylammonium chlorides is for compounds with 14 carbon atoms in the alkyl chain. The results show that QACs are generally more effective against Gram positive bacteria in comparison to Gram negative ones. QACs are positively charged compounds that are naturally attracted to negatively charged substances such as bacterial proteins essential for the structure and enzymatic activities of the cell. For the studied piperidine-based sulfobetaines there are no significant differences between results of MIC, as well as zones of inhibited growth of microorganism, for Gram positive and Gram negative bacteria. The similar effect on both the Gram positive and Gram negative microorganisms illustrates the preferred broad-spectrum activity of the compounds studied. The same effect was observed for series a of N-alkyl betaines (C8, C12, C16, C18) [30]. The MICs of the betaine series decreased with increasing chain length. The best microbiological activity was obtained for compounds with 16 carbon atoms, with MICs of 61 and 120 μM for *S. aureus* and *E. coli*, respectively. The MIC values obtained for the surfactants studied are very small. Ward *et al*. [12] evaluated this parameter for some sulfopropylbetaine copolymers. The lowest observed MIC value was 1.1 mg/mL for *E. coli* and 1.3 mg/mL for *S. aureus* [12]. Additionally, the antimicrobial activity of piperidine-based compounds was shown for 4-amidopiperidine-C12 (4AP12), the base form of 4-dodecaneamidopiperidine HCl [31]. Similar to surfactants obtained in this study, the 4AP12 has broad-spectrum activity against both Gram negative and Gram positive bacteria and fungi. Moreover, the authors of the cited work compared 4AP12 with its analogue with 16 carbon atoms in its acyl chain. The results showed comparable activity of both compounds against the tested microorganisms. For some bacterial and fungal strains the values of MIC were higher for 4AP16 (*A. baumannii*, *C. glabrata* ATCC CBS138), for some lower (*S. aureus* ATCC 25923). The possible antimicrobial mechanism of piperidine sulfobetaines is connected to their disruption of the cell membrane of microorganisms and subsequent cell lysis. The study suggests that all newly synthesized surfactants show antimicrobial activities, but the highest activity was determined for surfactants P16S4 and P16S3, i.e., those with the longest alkyl chain.

## Conclusions

The new sulfobetaine surfactants not only reduce the surface tension and form aggregates but also inhibit the growth of microorganisms even when applied in low concentrations. The surface properties, as well as antimicrobial activity of surfactants, dependent on their structure, the length of carbon chain and spacers between positively and negatively charged head groups. It was found that surfactants, both propane and butane derivatives, with the highest number of carbon in alkyl chain (P16S3 and P16S4) show the highest antimicrobial activities as well as the lowest CMC and highest efficiency of adsorption among all surfactants considered.
